# Laparoscopic sleeve gastrectomy in obese patients with ventricular assist devices: a data note

**DOI:** 10.1186/s13104-020-05272-2

**Published:** 2020-09-17

**Authors:** Adrian daSilva-deAbreu, Kiran Garikapati, Bader Aldeen Alhafez, Sapna Desai, Clement Eiswirth, Selim Krim, Hamang Patel, Carl J. Lavie, Hector O. Ventura, Juan Francisco Loro-Ferrer, Stacy A. Mandras

**Affiliations:** 1 The John Ochsner Heart and Vascular Institute, Ochsner Clinic Foundation, New Orleans, LA USA; 2 Ochsner Clinical School, Faculty of Medicine, The University of Queensland, New Orleans, LA USA; 3 Doctoral School, Universidad de Las Palmas de Gran Canaria, Las Palmas, Spain; 4 Department of Internal Medicine, The Ohio State University, Columbus, OH USA

**Keywords:** Heart failure, Ventricular assist devices, Heart-assist devices, Bariatric surgery, Laparoscopic sleeve gastrectomy, Weight loss, Body mass index, Heart transplantation, Metabolism, Echocardiography

## Abstract

**Objectives:**

Patients with end-stage heart failure (ESHF) treated with ventricular assist devices (VADs) tend to gain weight after implantation, which is associated with higher complication rates and is a contraindication for heart transplantation (HT). The objective was to analyze the outcomes of obese patients with ESHF and VADs who underwent laparoscopic sleeve gastrectomy (LSG) at Ochsner Medical Center in New Orleans, which is the only program performing VADs and HT in the State of Louisiana, and also one of the largest VAD centers in the USA.

**Data description:**

This dataset contains detailed baseline, perioperative, and long-term data of patients with VADs undergoing LSG. These variables were collected retrospectively from electronic medical records. Patients who achieved ≥ 50% excess BMI loss, BMI ≤ 35 kg/m^2^, listing for HT, HT, or myocardial recovery were identified and the timing to each of these milestones was documented. These data can be used alone or in combination with other datasets to achieve a larger sample size with more power for further analysis of these variables, which include the most important, standard, and objective bariatric and ESHF outcomes of patients with VADs undergoing LSG. Elaboration of composite outcomes is feasible.

## Objective

The use of ventricular assist devices (VADs) in patients with end-stage heart failure (ESHF) has grown significantly over the last decade. Nowadays, many patients undergo VAD implantation as a bridge to heart transplantation (HT), and remain on VAD support while awaiting availability of a healthy compatible heart. In other cases, patients undergo VAD implantation after being rejected for HT due to significant obesity (BMI ≥ 35 kg/m^2^) [1].

After VAD implantation, patients tend to feel better with appetite improvement, leading to increased caloric intake. Although exercise tolerance often improves too, it remains limited when compared to healthy subjects [2]. These factors, in addition to other comorbidities like depression, lead to weight gain after VAD implantation, especially in those patients with BMI < 35 kg/m^2^ [3]. The latter situation could lead to many obese patients with VADs as bridge to HT being deemed unsuitable candidates once they reach a BMI ≥ 35 kg/m^2^. Furthermore, obesity among patients with VADs has been associated with higher complication rates [4].

Bariatric surgery is an option for patients with VADs to lose weight and improve HT candidacy, with some studies reporting promising results [5–7]. Over the last years, laparoscopic sleeve gastrectomy (LSG) has become the preferred bariatric surgery for obese patients with VADs due to its effectiveness and safety profile [8]. However, postoperative complications may be more common in these patients due to their need for chronic anticoagulation and comorbidities.

The objective was to analyze the outcomes of obese patients with ESHF and VADs who underwent LSG at Ochsner Medical Center in New Orleans, the only VAD and HT program in the State of Louisiana, and also one of the largest VAD centers in the USA. This dataset was used in a study submitted for publication somewhere else [9].

## Data description

These data were collected retrospectively from the electronic medical records of obese patients with ESHF who underwent LSG at Ochsner Medical Center in New Orleans. All 8 patients were 18 years or older (Table 1)

**Table 1:** Overview of data files/datasets

Label	Name of data file/data set	File types (file extension)	Data repository and identifier (DOI or accession number)
Data file 1	Database LSG in patients with VADs	xlsx (or Excel)	Mendeley https://dx.doi.org/10.17632/8s35tzr33g.2
Data file 2	Description of variables	xlsx (or Excel)	Mendeley https://dx.doi.org/10.17632/8s35tzr33g.2

Baseline variables include demographics, VAD type, anthropometric parameters (BMI, excess BMI, percentage of excess BMI, weight at VAD implantation and LSG, etc.), echocardiographic parameters of left and right ventricular functions, New York Heart Association Functional Class (NYHA FC), and blood test variables, including B-natriuretic peptide, lactate dehydrogenase, and fasting lipid panel data.

Anthropometric data were also obtained at multiple timepoints during follow-up. Postoperative echocardiographic data correspond approximately to 6 months after LSG, while blood test and NYHA FC data were collected around the 3-month follow-up. Almost all data were obtained during euvolemic state (compensated ESHF), except for the postoperative NYHA FC data of patient 8, who had a mild ESHF decompensation.

Perioperative coagulation data were collected, including the most recent international normalized ratio (INR) and partial thromboplastin time (PTT) prior to LSG, the highest INR and PTT during the 48 h post-LSG, postoperative transfusions, hospital length of stay, intensive care unit length of stay, and multiple adverse outcomes. The numbers of hospital admissions and of hospitalization days for the 6-month periods prior to and after LSG, respectively, were also collected.

Patients who achieved ≥ 50% excess BMI loss, BMI ≤ 35 kg/m^2^ (appropriate BMI for HT), listing for HT, HT, or myocardial recovery were identified and timing to each of these milestones was documented. A decrease of 50% of excess BMI is conventionally considered a successful outcome after LSG, while the other outcomes are of major relevance from an ESHF and HT standpoint.

Data files 1 and 2 contain the complete dataset and a description of each variable, respectively. These data show the most important outcomes and efficacy of LSG in obese patients with VADs to improve their candidacy for HT as well as other major bariatric and ESHF parameters.

In patients with VADs, LSG is only performed in carefully selected patients in few centers for ESHF and HT. Hence, the sample sizes of published studies in this topic range from 1 to 11 patients [5, 10]. These data can be combined with other datasets to create multicenter cohorts to increase power and external validity of future studies evaluating the effects of LSG on BMI/weight loss, echocardiographic, functional, metabolic, and more importantly, ESHF outcomes such as listing for HT, HT, and myocardial recovery. The objective and standard definition of these variables across studies, and their relevance for clinical management, make dataset combinations feasible, even for comparing outcomes between patients with VADs undergoing LSG vs. patients who receive VAD implantation and LSG simultaneously.

Future studies could analyze composite outcomes including reaching a BMI ≤ 35 kg/m^2^, which is indicative of enough decrease in BMI as to improve their candidacy for HT, in combination with traditional ESHF outcomes such as listing for HT, HT, or myocardial recovery. Additionally, adverse events and other negative outcomes could also be analyzed as composite.

## Limitations

This is a single-center database, hence, the generalizability of the information it provides should be interpreted cautiously, and attention should be paid to the baseline characteristics and perioperative management, which has been described above and in the original research study published by our team using this dataset.

Although this is one of the largest datasets of obese patients with VADs who have undergone any bariatric surgery, its sample size is not large enough to allow for more advanced inferential statistical analysis such as regressions. However, considering that bariatric and ESHF outcomes are very standard among studies, it is feasible to combine this dataset with other single or multicenter samples to achieve a higher statistical power for more advanced statistical tests that would allow to identify predictors of successful and poor outcomes, which will help to guide clinical practice and improve quality of care for these complex patients with delicate health.

## Data Availability

The data described in this Data note can be freely and openly accessed on Mendeley Data under https://doi.org/10.17632/8s35tzr33g.2. Please see Table 1 and Refs. [9, 11] for details.

## Abbreviations

BMI: Body mass index
ESHF: End-stage heart failure
HT: Heart transplantation
INR: International normalized ratio
LSG: Laparoscopic sleeve gastrectomy
NYHA FC: New York Heart Association functional class
PTT: Partial thromboplastin time
VAD: Ventricular assist device
